# An Evaluation of the Gap Sizes of 3-Unit Fixed Dental Prostheses Milled from Sintering Metal Blocks

**DOI:** 10.1155/2017/7847930

**Published:** 2017-01-29

**Authors:** Jae-Kwan Jung

**Affiliations:** Department of Dental Technology, Daejeon Health Institute of Technology, 21 Chungjeong-ro, Dong-gu, Daejeon 34504, Republic of Korea

## Abstract

This study assessed the clinical acceptability of sintering metal-fabricated 3-unit fixed dental prostheses (FDPs) based on gap sizes. Ten specimens were prepared on research models by milling sintering metal blocks or by the lost-wax technique (LWC group). Gap sizes were assessed at 12 points per abutment (premolar and molar), 24 points per specimen (480 points in a total in 20 specimens). The measured points were categorized as marginal, axial wall, and occlusal for assessment in a silicone replica. The silicone replica was cut through the mesiodistal and buccolingual center. The four sections were magnified at 160x, and the thickness of the light body silicone was measured to determine the gap size, and gap size means were compared. For the premolar part, the mean (standard deviation) gap size was nonsignificantly (*p* = 0.139) smaller in the SMB group (68.6 ± 35.6 *μ*m) than in the LWC group (69.6 ± 16.9 *μ*m). The mean molar gap was nonsignificantly smaller (*p* = 0.852) in the LWC (73.9 ± 25.6 *μ*m) than in the SMB (78.1 ± 37.4 *μ*m) group. The gap sizes were similar between the two groups. Because the gap sizes were within the previously proposed clinically accepted limit, FDPs prepared by sintered metal block milling are clinically acceptable.

## 1. Introduction

At present, all-ceramic crowns that do not contain metal are preferred for dental restorations, but metal-ceramic crowns (porcelain fused to metal crown, PFM) remain widely used as fixed dental prostheses (FDPs) [1, 2]. Gap is one of the most important factors related to the oral lifespan of FDPs [3]. Fitness refers to the gap between abutments and the FDPs. When the gaps are large, there is a much higher chance of food residue and plaque accumulation than when the gaps are small, which increases the incidence of secondary caries [4]. Therefore, when the gaps are small, the oral lifespan of the FDPs is extended.

There is a wide array of factors which can affect gap sizes. This includes the 25 *µ*m cement space required for fixing FDPs onto abutments [5], which is suggested as the ideal value according to specification number 96 of the American Dental Association (ADA) [6]. In addition, there are many variables that affect the gap, such as the materials used, the techniques in which the materials are used, the competency of the operator, the area and method of gap measurement, and abutment design. The clinically allowable gap value remains debated, but many researchers are suggesting that a gap of 120 *µ*m is acceptable [7].

Gaps of PFM are dependent on the metal cores. Alloys are commonly utilized as a substructure material, and the lost-wax technique and casting method proposed by Taggart in 1907 are mainly used in FDP preparations containing alloys [8]. This method is sensitive but possesses numerous limitations and requires time-consuming manual preparation for all procedures.

Currently, a variety of dental computer aided design-computer aided manufacturing (CAD-CAM) systems have been commercialized for preparation of prostheses, using diverse materials. By utilizing such systems and materials, many preparation steps can be omitted and the process can be simplified as compared to conventional preparation methods. Currently, ceramic and metal crowns using dental CAD-CAM systems are widely used with the fabrication of ceramic crowns using a subtractive method. Comparative analyses of the gaps between ceramic crowns fabricated through such method and other conventional methods have been reported in a number of studies [9–11]. Few studies reported larger gaps in ceramic crowns fabricated by CAD-CAM techniques [9, 10], but the size of the gap remained within the clinically acceptable range.

Hard metal block manufacturing, using the subtractive method, or melting of metal powder through selective laser sintering (SLS) while using an additive has been introduced in dental CAD-CAM system-assisted preparation of FDPs with metal materials [3]. Various studies have assessed gaps of completed FDPs, using these two methods [12–15].

Earlier studies [13, 16] have reported that fabrication by the SLS produced larger gaps than did fabrication by the lost-wax technique. Moreover, the studies that evaluated the gaps of FDPs fabricated by milling hard metal blocks indicated that the marginal gap was smaller than that obtained when using conventional fabrication methods [17], whereas another study reported that conventional methods produced smaller marginal gaps [14]. The reason for such conflicting results may be attributed to differences in the specimens used.

In recent years, sintering metal block manufacturing via a subtractive method was introduced as a novel FDP preparation method. This method is almost identical to that previously used for hard metal block manufacturing. However, given their hardness, hard metal blocks are difficult to manufacture, while sintering metal blocks possess physical properties that are more similar to those of hard wax blocks than those of hard metal blocks. Thus, sintering metal blocks are convenient to manufacture and can be cut using a small processor. The material undergoes sintering after manufacturing and thereafter retains its original physical properties.

Considering the physical properties of the material, simple trimming is possible prior to sintering. Because the manufacturing is based on a dry process, no additional time is required to remove coolant, and there is less risk of material contamination. Accordingly, overload of the manufacturing devices in the CAD-CAM system is reduced, which therefore prolongs the lifespan of the tools, such as burs, and shortens the manufacturing time as compared to that required for hard metal blocks.

Despite the multiple advantages described above, gap assessments of FDPs fabricated via manufacturing of sintering metal blocks have not yet been performed. Therefore, this study aimed to evaluate the clinical acceptability of FDPs that were prepared by sintering metal blocks, based on fitness.

## 2. Materials and Methods

### 2.1. Study Model Preparation

**Figure 1:**
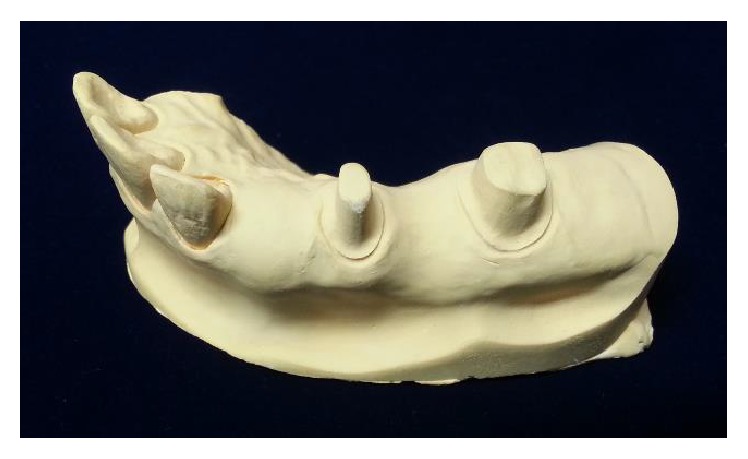
Main model.

In this study, a main model, consisting of a missing maxillary right 2nd premolar with maxillary right 1st premolar and 1st molar abutments, was prepared (Model #3017, Viade Products, Inc., Camarillo, CA, USA). The abutments were removed at a depth of 1.2 mm, and a margin was created in a 360° deep chamfer form, with an angle of 12°. After formation of the abutments, individual trays were prepared with dental resins (Trayplast, Vertex Pharmaceuticals B.V., Haarlem, Netherlands) (Figure 1), using this model. A total of 10 individual trays were prepared, and 10 impressions were obtained with silicone (Fresh, Dreve Dentamid GmbH, Unna, Germany) based on the main model. Ten study models were then prepared by injecting dental epoxy into the impression (Modralit® 3K, Dreve Dentamid GmbH, Unna, Germany).

### 2.2. Digital Model Preparation and 3-Unit FDP Design

To develop study groups from the 10 prepared study models, a digital model was prepared with a dental scanner (D-700, 3Shape A/S, Copenhagen, Denmark). The scanning procedure was as follows. A study model was firmly fixed onto the scanning holder. Once the scanning commenced, the laser read the model information in the form of lines, and a camera inside the scanner obtained images of the lines in the model.

Exclusive dental software (3Shape Dental Designer, 3Shape A/S, Copenhagen, Denmark) was used to process the images of model lines by triangulation, which were rearranged based on the final information, yielding three-dimensional (3D) digital models of the study model. In order to prepare sensitive digital models, multiple scans were made of each model. The entire model was exposed to the scan first, and each abutment was then scanned, individually. The images that were obtained from each scan were constructed into a total of 10 digital models through overlaps, using the dedicated dental software (3Shape Dental Designer, 3Shape A/S, Copenhagen, Denmark).

Using the completed digital model, an experienced technician designed 3-unit FDPs with the dental software (3Shape Dental Designer, 3Shape A/S, Copenhagen, Denmark) in a core form for preparation of the metal-ceramic crown. The thickness of the specimens was set at 0.5 mm in accordance with the program specification, the internal cement thickness for adhesion was set at 30 *μ*m, at a distance of 0.5 mm from the upper margin line, and the area of the connector was 9 mm^2^. The completed design information was saved as 10 standard template library (STL) files. An STL file is a file for open use across various dental CAD-CAM devices.

### 2.3. Preparation of Sintering Metal-Assisted 3-Unit FDPs

To prepare the experimental groups based on STL files of the completed specimen design, the STL file was implemented in a dedicated program (Ceramill Mind, Amann Girrbach AG, Koblach, Austria). After the implementation, the path and margin were reviewed prior to manufacturing. Based on the information of the reviewed FDPs, milling was performed with a manufacturing device using the subtractive method (Ceramill Motion 2, Amann Girrbach AG, Koblach, Austria) and a sintering metal block (Ceramill Sintron 71 XXS [10 mm], Amann Girrbach AG) comprised of a cobalt-chromium alloy. The composition of the sintering metal is described in Table 1.

**Figure 2:**
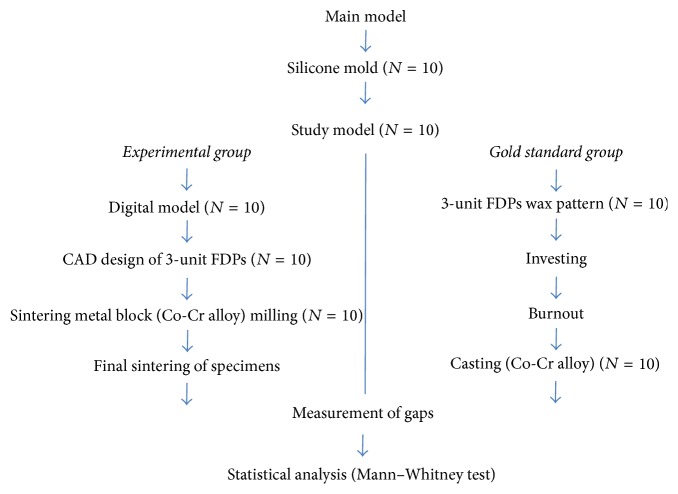
Work flow of study.

Sintering metal blocks have mechanical properties similar to wax blocks and require a sintering process after milling, as it is soft prior to sintering. Milling specimens were placed in the dedicated box (Ceramill Sinter box, Amann Girrbach AG) of the furnace (Ceramill Argotherm, Amann Girrbach AG) and then sintered. The sintering box was filled with argon gas during sintering to prevent oxidation of the specimens. Sintering was performed at 1,280°C, and the temperature then gradually decreased over a period of 5 h. The mechanical properties of the metal after final sintering are shown in Table 2. After completing the sintering, these specimens were used for the experimental group (SMB group) (Figure 2).

### 2.4. Preparation of Lost-Wax Technique and Casting Method-Assisted 3-Unit FDPs

To prepare the control group (LWC group) for comparison with the SMB group, 3-unit FDPs were constructed using the lost-wax technique and casting method on the same models used for preparation of the experimental group. The same conditions were used as for the experimental group, but a die spacer was applied 4 times to the abutments that were located 0.5 mm away from the margin line. Previous studies have reported that this results in a gap of 30 *μ*m thickness [18, 19].

After die-spacer application, dental wax was used to generate wax patterns of the 3-unit FDPs. Margin lines were then confirmed under a microscope (AIS-10L, Daemyung Optical Product, Daejeon, Korea; 10x), and the 10 completed wax patterns were subsequently subjected to spruing, investing, and burnout, using a standard methodology. After the burnout, the patterns were used for casting with a cobalt-chromium alloy (Wirobond®C, BEGO GmbH, Bremen, Germany) to prepare the 10 controls (LWC group) (Figure 2). The composition of the used alloy is shown in Table 1.

### 2.5. Gap Measurements

**Figure 3:**
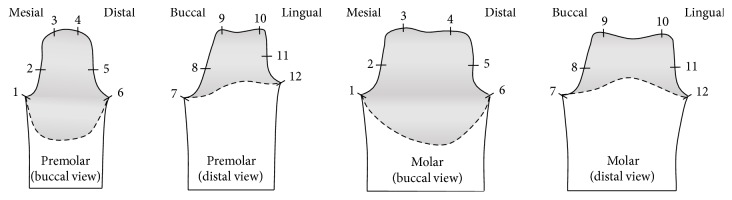
Measurement positions of gap of abutments (margin part: P1, P6, P7, and P12; axial wall part: P2, P5, P8, and P11; occlusal part: P3, P4, P9, and P10).

The gap measurements were selected as described in Figure 3 [20]. Twelve points were measured per abutment, and fitness was measured for a total of 480 times, with 24 points per specimen and 20 specimens in the two groups. A gap was defined as the vertical distance from the abutment models to the specimens for all of the measurement parts [20, 21]. The 12 points that were measured in each abutment were categorized into a margin part (1, 6, 7, and 12), axial wall part (2, 5, 8, and 11), and occlusal part (3, 4, 9, and 10) (Figure 3).

For the gap assessment method, a silicone replica technique that had been verified in previous studies was used [22, 23]. This method replicates the distance between the specimens and the model with light body silicone, and the gap is then measured based on silicone thickness. In accordance with this method, light body silicone (Fresh, Dreve Dentamid GmbH, Unna, Germany) was used to fill the inside of the specimen, and pressure was then applied to the study models. In this study, pressure (about 50 N) was applied to the abutment models towards the tooth axis [14, 15], and this was maintained until the light body silicone had completely polymerized.

**Figure 4:**
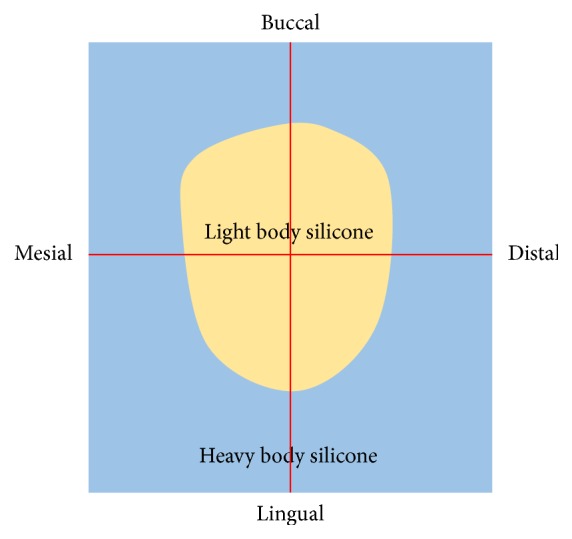
The silicone replica sectioned twice (red line).

**Figure 5:**
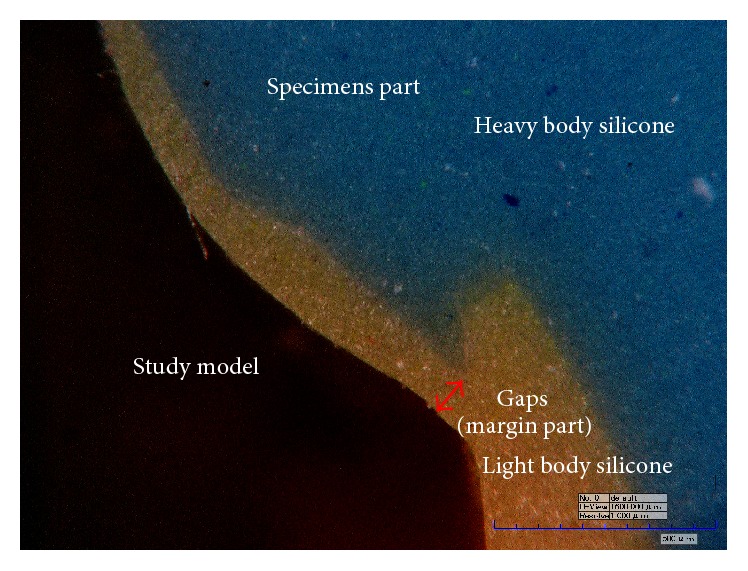
Measurement of gaps by a digital microscope (×160 magnification).

Once the light body silicone had completely polymerized, the specimen was removed carefully, and the light body silicone was reinforced by heavy body silicone (Fresh, Dreve Dentamid GmbH). Such reinforcement was done because delicate cutting and measurement are difficult for thin light body silicone. When heavy body silicone is polymerized (Figure 4), the silicone replica that was obtained was divided into four equal parts by cutting through the center of the buccolingual and mesiodistal dimensions, as shown in Figure 4. The divided silicone replica was then subjected to gap measurement under a 160x calibrated digital microscope (KH-7000, HIROX, Hackensack, NJ, USA) (Figure 5).

### 2.6. Statistical Analysis

The gaps measured in the SMB group and LWC group were statistically compared using the nonparametric Mann–Whitney test (significance level: 95%). All statistical analyses were performed with IBM SPSS, version 20 (IBM Corporation, Armonk, NY, USA).

## 3. Results

### 3.1. Premolars

The means and standard deviations of the points that were measured on the 3-unit premolar FDPs that were prepared by the two different methods are described in Table 3. The Mann–Whitney test showed no statistically significant differences in the means between the two groups for P1, P6, P7, P9, and P12 (all *p* > 0.05), while significant differences were found in the means between the two groups for P2, P3, P4, P5, P8, P10, and P11 (all *p* < 0.05). The mean gap in all of the measurements regardless of points was slightly smaller in the SMB group (68.6 ± 35.6 *μ*m) than in the LWC group (69.6 ± 16.9 *μ*m), but this was not statistically significant (*p* = 0.139; Table 3).

After comparing each point, P1–P12 were divided into the margin part (P1, P6, P7, and P12), axial wall part (P2, P5, P8, and P11), and occlusal part (P3, P4, P9, and P10), and each part was compared; the means and standard deviations of the gaps that were measured for the three parts are shown in Table 4. Both the LWC group and the SMB group showed the smallest gap in the axial wall part and the biggest gap in the occlusal part. The margin part and axial wall part in the SMB group had smaller gaps than did those in the LWC group, but the occlusal part had a smaller gap in the LWC group than in the SMB group (Table 4).

When the mean gaps in the parts were compared between the two groups, the margin part showed no significant difference (*p* = 0.054), whereas the axial wall part and occlusal part were statistically significantly different (*p* < 0.05; Table 4).

### 3.2. Molar

The mean and standard deviation of each point that was measured on the molars of the two groups are presented in Table 3. There were no statistically significant differences in the means between the two groups for P1, P2, P6, P7, P8, P10, P11, and P12 (all *p* > 0.05), while significant differences were observed in the means between the two groups for P3, P4, P5, and P9 (all *p* < 0.05; Table 3). The mean gap in all of the measurement parts, P1–P12, was smaller in the LWC group (73.9 ± 25.6 *μ*m) than in the SMB group (78.1 ± 37.4 *μ*m), but this difference was not statistically significant (*p* = 0.852; Table 4).

Similar to the premolars, P1–P12 were divided into the margin part (P1, P6, P7, and P12), axial wall part (P2, P5, P8, and P11), and occlusal part (P3, P4, P9, and P10), and each part of the molar was compared between the two groups; the means and standard deviations of the gaps that were measured on the 3 parts are shown in Table 4.

Both the LWC group and the SMB group showed the smallest gap in the axial wall part and the biggest gap in the occlusal part. The margin part and axial wall part showed smaller gaps in the SMB group than in the LWC group, whereas the occlusal part was smaller in the LWC group than in the SMB group (Table 4).

The Mann–Whitney test was used to compare the means of the gaps in the parts between the two groups. The margin part did not differ significantly (*p* = 0.181), while the mean differences in the axial wall part and occlusal part were statistically significant (both *p* < 0.05; Table 4).

## 4. Discussion

This study aimed to evaluate the clinical acceptability of 3-unit FDPs that were prepared by sintering and milling metal block bases, a material that has recently been newly introduced, based on gap sizes. In this study, a total of 20 3-unit FDPs, including 10 specimens that were prepared by milling of sintering metal blocks and 10 specimens that were prepared by the conventional lost-wax technique and casting method, were prepared on the same model. Gap assessments were performed on 480 points in total, 24 points per specimen (abutments × 2), for 20 specimens.

Based on the mean of the overall gaps that were measured on both abutments, the gaps of the premolar and molar were confirmed to be slightly superior in the SMB group and the LWC group, respectively, but these differences were not statistically significant. Consequently, the FDPs that were prepared using sintering metal block manufacturing are believed to be clinically acceptable, based on gap size.

The limits for clinical acceptance in FDPs remain controversial, and many researchers have proposed that 120 *μ*m be used as cut-off [7]. McLean and von Fraunhofer [7] evaluated the fitness of 1,000 fixed prostheses older than 5 years and reported that a gap of at least 100 *μ*m is necessary to prevent clinical problems but that it should not exceed 120 *μ*m in order to retain clinical acceptability. Based on the clinical acceptance limit suggested by McLean, 3-unit FDPs that have been prepared by sintering metal block manufacturing are considered clinically acceptable.

To analyze fitness in greater detail, the present study further divided the various parts into the margin part, axial wall part, and occlusal part. The SMB group showed a superior gap in both the margin part and the axial wall part as compared to the LWC group, while the LWC group manifested superior fitness in the occlusal part as compared to the SMB group. Both groups exhibited the smallest gap in the axial wall part, whereas the largest gap was present in the occlusal part.

The LWC group manifested a larger gap in the occlusal part than in the margin and axial wall parts, but the gaps in the three parts were not markedly different. In contrast, the SMB group had a larger gap in the occlusal part than in the margin and axial wall parts. This may be explained as follows. Unlike the lost-wax technique, in which dental wax is melted and is prepared sequentially, blocks of material are used to manufacture the inside of the prostheses with a milling bur when using the milling method. This process is influenced by bur diameter, and it is therefore considered difficult to reproduce the connection from the axial wall to the occlusal part smoothly [14].

In addition, the larger gaps in the occlusal part than in the margin and axial wall parts in both groups are thought to be due to total occlusal convergence (TOC). A previous study has reported that the gap in the occlusal part in such prostheses is greatly influenced by larger TOC abutments [24]. In this study, 12° was used as the abutment TOC, and the above phenomenon seems to be improved as the angle is decreased.

There are several methods for assessing FDP gaps. First, specimens and models are smeared with dental epoxy, and cut sections are then confirmed, followed by gap measurement under a microscope [14, 25–28]. This method has a number of shortcomings, including specimen and model destruction and inconvenience, such as cutting after smearing. It is also impossible to measure all the various points after cutting. Second, marginal gaps are measured under a microscope, because the specimens are set on models [29, 30]. This method allows for measurements of various points, and specimens and models are not destroyed. However, internal gap measurements are not possible, and marginal gaps are measured only by vertical distance, so the accuracy of the measurement is relatively low.

The silicone replica technique that was used in the present study has been employed in many previous studies [3, 12, 14, 15], given its confirmed accuracy and reliability [22]. According to this method, light body silicone with replicated abutments and specimen gaps was cut, and, after confirming the cut section, the thickness of the light body silicone was measured under a microscope. This is a relatively simple, accurate method [22] that does not destroy the abutment and model, and it is the only method that can be used to measure the inside of the mouth. However, the amount of silicone used as well as shrinkage can affect the measurements, and 3D analyses are impossible as this is a 2D-based method. It is also limited to measurements of certain parts, because the measurements are done after cutting the silicone replica.

Örtorp et al. [14] measured the gaps of 3-unit FDPs that were prepared using hard metal block manufacturing with premolar and molar abutments. They found that gaps in the premolar margin part were in the range of 180–220 *μ*m, while in the molar margin part they were in the range of 236–260 *μ*m. Gaps in the axial wall part were 23 *μ*m and 30–33 *μ*m for the premolar and molar, respectively, and in the occlusal part they were 256–304 *µ*m for the premolar and 259–289 *µ*m for the molar; thus, the largest gap was in the occlusal part. This study was based on FDPs that were prepared through metal manufacturing, similar to that used in the present study, even though they were not prepared using sintering metal block manufacturing. However, the above study also showed a larger gap in the occlusal part than in the margin part and axial wall part, as found in this study.

The FDP gap is governed by diverse factors, including abutment shapes, materials used to obtain the impressions, the types of dental cement used, and the methods used for gap measurement. It is therefore difficult to determine FDP gaps accurately. In the present study, efforts were made to control such diverse variables, but assessment of gaps with FDPs only from the same case is a limitation of the study.

Accordingly, future studies need to investigate FDP gaps in a variety of cases and abutment shapes. Moreover, since sintering metal blocks are a newly developed material, diverse studies evaluating the clinical acceptability of FDPs made with this new material need to be performed.

## 5. Conclusions

In this study, sintering metal blocks were manufactured for the fabrication of 3-unit FDPs, which were then subjected to gap measurements. These fabricated specimens were then compared against those prepared using the conventional lost-wax technique and casting method, in order to assess the clinical acceptability of the sintering method, based on gap sizes.

There were no significant differences in the overall mean gap sizes that were measured on the specimens prepared using the two different methods. In addition, considering that the gaps of the 3-unit FDPs that were prepared by sintering metal block manufacturing did not exceed 120 *μ*m, which is the cut-off suggested by McLean, these FDPs are clinically acceptable. In the future, guidelines for suitable abutment shapes should be derived.

## Figures and Tables

**Table 1:** Composition of sintering metal and casting metal.

Component (%)	Sintering metal	Casting metal
Cobalt (Co)	66	61
Chrome (Cr)	28	26
Molybdenum (Mo)	5	6
Tungsten (W)	—	5
Silicon (Si)	<1	1
Cerium (Ce)	—	0.5
Iron (Fe)	<1	0.5
Niobium (Nb)	—	—
Manganese (Mn)	<1	—
Carbon (C)	—	<0.02

**Table 2:** Mechanical properties of sintering metal (after final sintering).

Tensile strength (Rm)	840 MPa
0.2% proof stress (Rp 0,2 )	450 MPa
Modulus of elasticity (E)	200 GPa
Elongation at rupture	20%
Vickers hardness	280 HV 10
CTE (25–500°C)	14.5*∗*10^−6^/K
Density	8.0 g/cm^3^
Open porosity	0%
Color	Silver
Oxide color	Grey-green
Alloy class	Type 4

**Table 3:** Mean ± standard deviation of each point of gap for two groups with results of the Mann-Whitney test (unit: *μ*m).

Points	*N*	Premolar			Molar		
		SMB^a^	LWC^a^	*p* value^b^	SMB^a^	LWC^a^	*p* value^b^
1	10	62.0 ± 22.7	59.4 ± 10.7	0.880	75.7 ± 36.8	98.0 ± 14.9	0.151
2	10	39.3 ± 17.6	55.6 ± 17.6	0.045	41.1 ± 15.0	48.7 ± 10.8	0.112
3	10	104.5 ± 21.0	79.3 ± 14.6	0.005	113.7 ± 20.1	82.7 ± 24.5	0.008
4	10	97.2 ± 17.8	79.0 ± 11.1	0.028	116.3 ± 20.0	75.8 ± 28.8	0.007
5	10	36.0 ± 12.3	50.0 ± 6.3	0.019	34.8 ± 8.3	59.0 ± 7.6	0.001
6	10	55.6 ± 21.3	68.1 ± 11.1	0.131	60.1 ± 29.1	76.2 ± 20.0	0.059
7	10	58.3 ± 22.0	71.4 ± 11.8	0.096	101.4 ± 36.0	74.2 ± 24.3	0.096
8	10	39.3 ± 15.1	62.3 ± 14.5	0.005	52.4 ± 12.0	52.0 ± 12.1	0.940
9	10	109.0 ± 36.2	86.6 ± 14.2	0.082	119.2 ± 29.1	83.4 ± 26.9	0.016
10	10	116.0 ± 33.6	90.2 ± 12.0	0.007	100.2 ± 17.3	102.7 ± 20.4	0.597
11	10	44.2 ± 11.4	63.6 ± 12.7	0.003	53.8 ± 17.2	51.3 ± 12.6	0.705
12	10	62.5 ± 22.6	70.0 ± 12.0	0.406	68.8 ± 28.5	83.4 ± 22.7	0.257

Total	120	68.6 ± 35.6	69.6 ± 16.9	0.139	78.1 ± 37.4	73.9 ± 25.6	0.852

^a^SMB: sintering metal block (experimental group); LWC: lost-wax technique + casting (gold standard group).

^b^The results of Mann-Whitney test.

**Table 4:** Mean ± standard deviation of locations of gap for two groups with results of the Mann–Whitney test (unit: *μ*m).

Locations	*N*	Premolar			Molar		
		SMB^a^	LWC^a^	*p* value^b^	SMB^a^	LWC^a^	*p* value^b^
Margin	40	60.0 ± 21.5	67.2 ± 12.0	0.054	76.5 ± 35.2	82.9 ± 22.1	0.181
Axial wall	40	40.0 ± 14.1	57.9 ± 14.0	0.001	45.5 ± 15.3	52.8 ± 11.2	0.006
Occlusion	40	106.7 ± 28.0	84.0 ± 13.4	0.001	112.4 ± 22.4	86.1 ± 26.4	0.001

^a^SMB: sintering metal block (experimental group); LWC: lost-wax technique + casting (gold standard group).

^b^The results of Mann–Whitney test.
